# Nanoplastics Cause Neurobehavioral Impairments, Reproductive and Oxidative Damages, and Biomarker Responses in Zebrafish: Throwing up Alarms of Wide Spread Health Risk of Exposure

**DOI:** 10.3390/ijms21041410

**Published:** 2020-02-19

**Authors:** Sreeja Sarasamma, Gilbert Audira, Petrus Siregar, Nemi Malhotra, Yu-Heng Lai, Sung-Tzu Liang, Jung-Ren Chen, Kelvin H.-C. Chen, Chung-Der Hsiao

**Affiliations:** 1 Department of Chemistry, Chung Yuan Christian University, Chung-Li 32023, Taiwan; sreejakarthik@hotmail.com (S.S.); gilbertaudira@yahoo.com (G.A.); 2 Department of Bioscience Technology, Chung Yuan Christian University, Chung-Li 32023, Taiwan; siregar.petrus27@gmail.com (P.S.); stliang3@gmail.com (S.-T.L.); 3 Department of Biomedical Engineering, Chung Yuan Christian University, Chung-Li 32023, Taiwan; nemi.malhotra@gmail.com; 4 Department of Chemistry, Chinese Culture University, Taipei 11114, Taiwan; lyh21@ulive.pccu.edu.tw; 5 Department of Biological Science & Technology, College of Medicine, I-Shou University, Kaohsiung 82445, Taiwan; 6 Department of Applied Chemistry, National Pingtung University, Pingtung 90003, Taiwan; 7 Center for Nanotechnology, Chung Yuan Christian University, Chung-Li 32023, Taiwan

**Keywords:** polystyrene, nanoplastics, neurotoxicity, behavior test, ecotoxicity, oxidative stress, zebrafish

## Abstract

Plastic pollution is a growing global emergency and it could serve as a geological indicator of the Anthropocene era. Microplastics are potentially more hazardous than macroplastics, as the former can permeate biological membranes. The toxicity of microplastic exposure on humans and aquatic organisms has been documented, but the toxicity and behavioral changes of nanoplastics (NPs) in mammals are scarce. In spite of their small size, nanoplastics have an enormous surface area, which bears the potential to bind even bigger amounts of toxic compounds in comparison to microplastics. Here, we used polystyrene nanoplastics (PS-NPs) (diameter size at ~70 nm) to investigate the neurobehavioral alterations, tissue distribution, accumulation, and specific health risk of nanoplastics in adult zebrafish. The results demonstrated that PS-NPs accumulated in gonads, intestine, liver, and brain with a tissue distribution pattern that was greatly dependent on the size and shape of the NPs particle. Importantly, an analysis of multiple behavior endpoints and different biochemical biomarkers evidenced that PS-NPs exposure induced disturbance of lipid and energy metabolism as well as oxidative stress and tissue accumulation. Pronounced behavior alterations in their locomotion activity, aggressiveness, shoal formation, and predator avoidance behavior were exhibited by the high concentration of the PS-NPs group, along with the dysregulated circadian rhythm locomotion activity after its chronic exposure. Moreover, several important neurotransmitter biomarkers for neurotoxicity investigation were significantly altered after one week of PS-NPs exposure and these significant changes may indicate the potential toxicity from PS-NPs exposure. In addition, after ~1-month incubation, the fluorescence spectroscopy results revealed the accumulation and distribution of PS-NPs across zebrafish tissues, especially in gonads, which would possibly further affect fish reproductive function. Overall, our results provided new evidence for the adverse consequences of PS-NPs-induced behavioral dysregulation and changes at the molecular level that eventually reduce the survival fitness of zebrafish in the ecosystem.

## 1. Introduction

Approximately 300 million tons of plastic waste is being produced every year, which is equivalent to the weight of the entire human population. If the current trend continues, by 2050 the plastic industry could account for 20% of the world’s total oil consumption. Only 9% of plastic waste ever produced was recycled and 12% was incinerated, while the remaining 79% accumulated in landfills, dumps, or the natural environment [1]. However, plastic waste whether in a river, an ocean, or on land, can persist in the environment for centuries. Plastic debris can come in all shapes and sizes. Macroplastic is relatively large particles of plastic (typically more than about 5 mm) found, especially in the marine environment. Those that are less than five millimeters in length are called microplastics. Macroplastic is clearly visible plastic that can be caught and generally will not have a direct impact on the food chain. Farm animals or fish that mistake them for food swallow the majority of these tiny plastic particles and, thus, can find their way into the human body [2].

However, the potential risks that are associated with microplastics exposure are unknown for both wildlife and humans. Critical information—long/short mode of exposure and effects data needed for the risk assessments—are still lacking. Human populations could be exposed to microplastics directly from the food or the environment. Multiple studies have documented that microplastics are globally dispersed in marine sediments, oceans, and shorelines in the form of debris that is generated from the degradation/hydrolysis or buoyant Styrofoam of plastics [3,4]. Microplastics can disrupt the food biota and environmental health due to their poor degradation capability and small size [5,6]. Likewise, the toxic effects of smaller sized nanoplastics are also very plausible, however, there are limited relevant data and their research is in its infancy [7,8]. Thus far, it has been demonstrated that microplastics can be ingested and accumulated by small aquatic organisms, such as zooplankton (*Daphni*a *magna, Centropages typicus*) and shrimp (*Crangon cargon*), subsequently being transferred to the large organism, such as sea turtles [9,10]. Another recent study on water flea (*Ceriodaphinia dubia*) reported that the acute and chronic exposure of polyester fibers resulted in significant effects on reproduction and survival [11]. In fish, microplastics have been found to cause major adverse effects, including hepatic stress, intestinal damages, and oxidative stress, among others [12,13,14]. Moreover, Karami et al. also found that the potential presence of microplastics in dried fish tissue: viscera and gills [15]. In embryos and adult fish, microplastics have been reported translocating from the digestive tract to gills and liver of zebrafish [12]. Together, these studies suggested that microplastics could exhibit toxicity in living organisms, the challenge could be extensive due to ubiquity in the environment and the translocation of potentially moving particles to animal body parts, usually consumed by humans.

The most abundant microplastics that are found in marine waters are low-density polyolefins and polystyrene [16,17]. Microplastics can be ingested by organisms and accumulated for a long time because of their tiny size and poor biodegradability. It was reported that microplastics can induce reactive oxygen species (ROS) formation and oxidative stress in mussels and lugworms [18,19]. In addition, microplastics can also induce the regulation of phagocytic activity of immune cells in worms [19]. However, the mechanism of the toxicity of microplastics is unclear and it needs further investigation. Enzymatic analysis and histology are widely used to determine the toxic effects of microplastics [20]. In all of these studies, the major factor was set on oral ingestion of microplastics, and only a few pieces of researches included other modalities for microplastic exposure. A study reported that, upon exposure, clean microspheres were not only found in the intestinal tract but also in the gills of the shore crab (*Carcinus maenas*) after 21 days of exposure [21]. Nanoplastics have an enormous surface area, bearing the potential to bind even bigger amounts of toxic compounds than microplastics.

Currently, very little data are available regarding the effects of nanoplastics on the development of aquatic organisms, whereas most of the studies have focused on the chemical aspects of nanoplastics. Indeed, the toxic effects of leachate from particles have been analyzed in different organisms, such as sea urchins [22] and mussels [22], whereas the behavioral and physical effects are still poorly explored [23]. To fill this gap, we evaluated the effects of environmentally relevant concentrations of nanoplastics on adult zebrafish behavior and biochemistry to assess the ecological toxicity of polystyrene nanoplastics. Polystyrene is a major kind of plastic of high production volume as well as the primary components of plastic debris contaminating the environment [24]. So far, no evidence exists regarding the potential effect of polystyrene nanoplastics on adult zebrafish behavior. In the present study, adult zebrafish were used to investigate the neurobehavioral changes that are induced by polystyrene nanoplastics and their distribution and accumulation in various tissues. Neurotransmitter amounts and enzyme activities for wither dopamine (DA), acetylcholine (ACh), serotonin (5-HT), melatonin (MT), γ-aminobutyric acid (GABA), Cytochrome P450, family 1, subfamily A, polypeptide 1 (CYP1A1), reactive oxygen species (ROS), or creatinine kinase (CK) contents were quantified to explore the toxicity mechanism.

## 2. Results

### 2.1. Overview of Experimental Design

**Figure 1:**
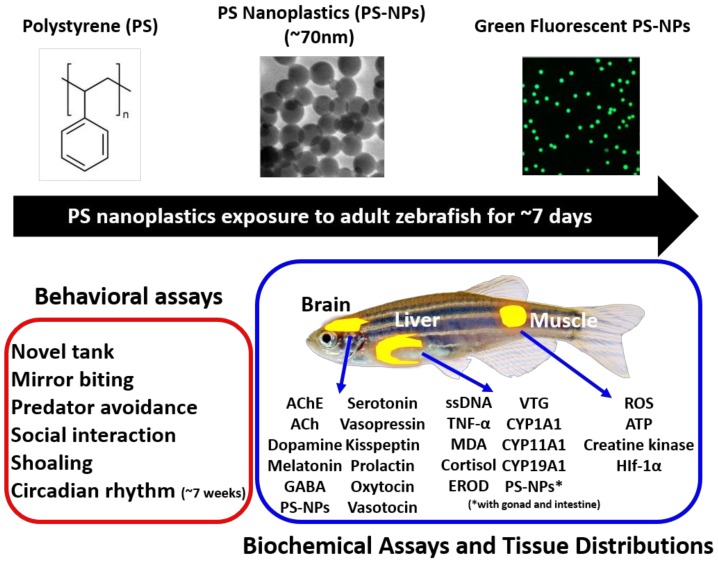
The experimental design to evaluate the ecotoxicity of polystyrene nanoplastics (PS-NPs). The chemical structure and particle size of polystyrene used in this study were summarized in the upper panel. The behavioral toxicity assays for PS-NPs were summarized in the left-right panel (red color). The biochemical endpoints for PS-NPs toxicity were summarized in the right lower panel (blue color).

A schematic representation of biological responses in zebrafish in response to polystyrene nanoplastics (PS-NPs) exposure is presented in Figure 1 based on data from this study. Figure 1 shows the shape and size of PS-NPs, as confirmed by transmission electron microscopy (upper panel). The diameter of PS-NPs used in this study was estimated to be around 70 nm. In addition, the Fluorescein isothiocyanate (FITC)-conjugated PS-NPs was used to trace the tissue distribution after waterborne exposure. The adult zebrafish aged six to seven months old were incubated with PS-NPs at doses of 0.5 and 1.5 ppm for around seven consecutive days and five behavioral tests (novel tank, mirror biting, predator avoidance, social interaction, and shoaling) were performed to measure its consequent behavioral alterations (lower left panel, highlighted in red). In addition, circadian rhythm locomotion activity test was performed after ~7 weeks incubation of 5 ppm PS-NPs to observe the long-term effect of high dose PS-NPs on zebrafish sleep/wake behavior. Finally, after all of the behavioral tests, excluding circadian rhythm locomotion activity test, were done, zebrafish were sacrificed and the brain, the liver, and the muscle tissues were dissected for biochemical marker measurement (lower right panel, highlighted in blue). For PS-NPs distribution, the brain, liver, male gonad, and intestine were collected and subjected to indirect PS-NPs quantification based on green fluorescence signal intensity.

### 2.2. PS-NPs Exposure Reduced Average Speed and Exploration Behavior in Zebrafish

**Figure 2:**
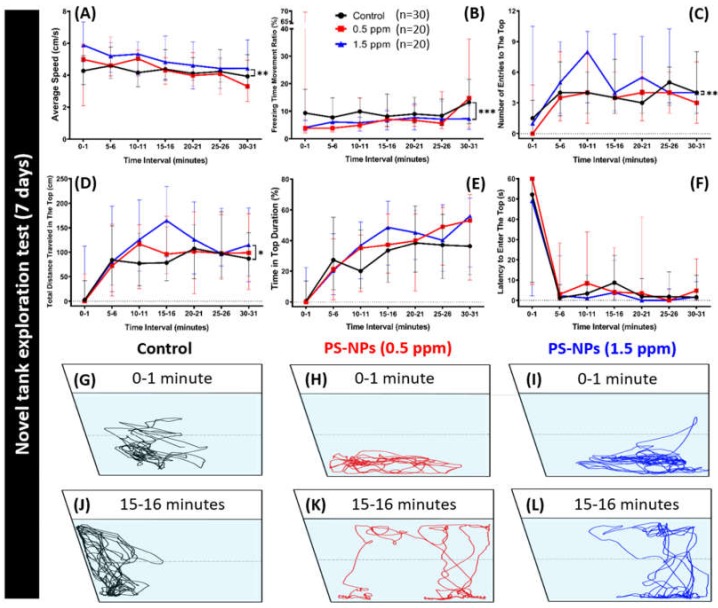
Novel tank behavior endpoints comparisons between control and polystyrene nanoplastics (PS-NPs)-exposed zebrafish groups after a ~7-day exposure. (**A**) Average speed, (**B**) freezing time movement ratio, (**C**) number of entries to the top, (**D**) total distance traveled in the top, (**E**) time in top duration, and (**F**) latency to enter the top were analyzed. The 1 min. locomotion trajectories for the control, 0.5 and 1.5 ppm PS-NPs exposed fish in the novel tank test were presented in (**G** to **L**), respectively. The black line represents the control group, the red line represents the low concentration PS-NPs group (0.5 ppm), and the blue line represents the high concentration PS-NPs group (1.5 ppm). The data are expressed as the median with interquartile range and were analyzed by a Kruskal–Wallis test, which continued with Dunn’s multiple comparisons test as a follow-up test (*n* = 30 for control; *n* = 20 for each PS-NPs-exposed group; * *p* < 0.05, ** *p* < 0.01, *** *p* < 0.001).

The locomotion activity and exploratory behavior were evaluated while using a novel tank test in PS-NPs-treated fishes at two different concentrations. Novel tank test is a behavior test to assess the zebrafish exploratory nature. This test exploits the natural tendency of zebrafish to initially diving to the bottom part of a novel tank, with a gradual increase in vertical activity over time. Typically, when zebrafishes are moved to a new environment, they display high anxiety and bottom-dwelling behavior, but they will start to explore the tank and move towards the upper area of the tank with anxiety relief once they adapt to the new environment (Figure 2G,J) [25]. In this test, we measured six important zebrafish behavior endpoints representing their swimming activity and exploratory behavior. The result showed that adult fish that were exposed to 0.5 ppm of PS-NPs showed no difference in their swimming activity when compared to the controls while hyperactivity-like behavior shown by the 1.5 ppm concentration group, supported by the high level of average speed and low level of freezing time movement ratio (Figure 2A,B). Furthermore, alteration in their exploratory behavior was observed in the high concentration of the PS-NPs-treated group. This abnormality was indicated by an increase in the number of entries in the top and total distance traveled in the top (Figure 2C,D,I,L), even though there was no significant difference shown in the time in top duration and latency to enter the top endpoints (Figure 2E,F). On another hand, this behavior alteration was not exhibited by the low concentration group fish (Figure 2C,D,H,K). Figure 2G–L and Supplementary Video S1 display the locomotion trajectories and behavioral changes for the control, 0.5 and 1.5 ppm PS-NPs exposed fish in the novel tank test.

### 2.3. PS-NPs Exposure Reduced Aggression and Predator Avoidance in Zebrafish

**Figure 3:**
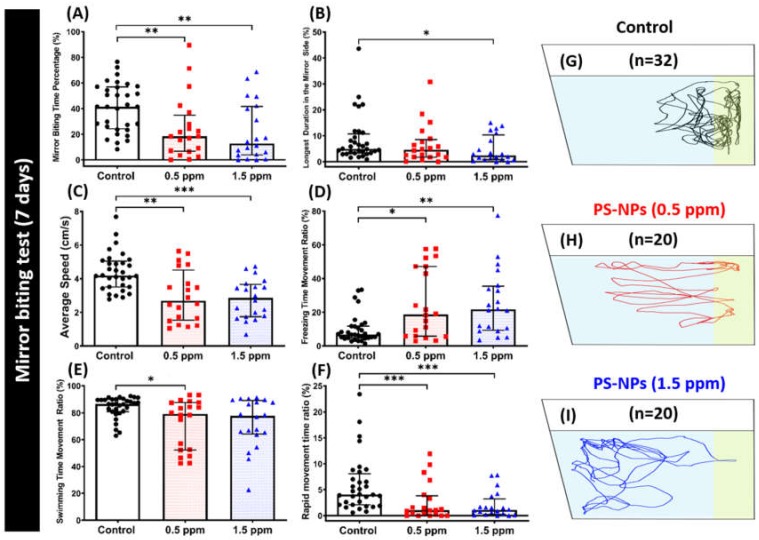
Mirror biting behavior endpoints comparisons between control and polystyrene nanoplastics (PS-NPs)-exposed zebrafish groups after a ~7-day exposure. (**A**) Mirror biting time percentage, (**B**) longest duration in the mirror side, (**C**) average speed, (**D**) freezing time movement ratio, (**E**) swimming time movement ratio, and (**F**) rapid movement time ratio were analyzed for mirror biting assay. The 1 min. locomotion trajectories for the control, 0.5 and 1.5 ppm PS-NPs-exposed fish in mirror biting tests were presented in **G** to **I**, respectively, with the yellow-colored zone as the mirror biting region. The data are expressed as the median with interquartile range and were analyzed by a Kruskal–Wallis test, which continued with Dunn’s multiple comparisons test as a follow-up test (*n* = 32 for control; *n* = 20 for each PS-NPs-exposed group; * *p* < 0.05, ** *p* < 0.01, *** *p* < 0.001).

A mirror biting test was conducted to determine the aggressive nature of zebrafish altered by the exposure of PS-NPs. The mirror biting assay is a simple and efficient method to test fish aggressiveness in terms of the frequency of the tested fish to bite their mirror images [26]. This test might also indicate a more general measure of social motivation or the intent to interact with a social partner [27]. In this test, chronic exposure of PS-NPs in both concentrations significantly reduced zebrafish aggressiveness. This phenomenon is shown by a decrease in the mirror biting time percentage and in the longest duration in the mirror side (Figure 3A,B). Interestingly, both concentrations of PS-NPs exposure reduced their locomotion behavior, as indicated by a decrease in the average speed, swimming time movement ratio, and rapid movement ratio, and an increase in the freezing time movement ratio as compared to the control group (Figure 3C–F). This result suggests that both 0.5 and 1.5 ppm of PS-NPs exposure reduce the aggressiveness in zebrafish. Figure 3G–I and Supplementary Video S2 display the locomotion trajectories and behavioral changes for the control, 0.5 and 1.5 ppm PS-NPs-exposed fish in the mirror biting test.

**Figure 4:**
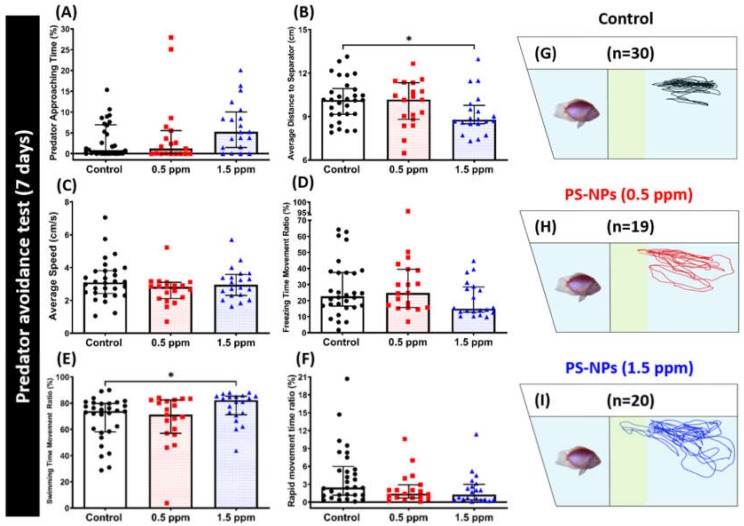
Predator avoidance behavior endpoints comparisons between control and polystyrene nanoplastics (PS-NPs)-exposed zebrafish groups after a ~7-day exposure. (**A**) Predator approaching time percentage, (**B**) average distance to the separator, (**C**) average speed, (**D**) freezing time movement ratio, (**E**) swimming time movement ratio, and (**F**) rapid movement time ratio were analyzed. The 1 min. locomotion trajectories for the control, 0.5 and 1.5 ppm PS-NPs exposed fish in the predator avoidance test were presented in **G** to **I**, respectively, with the yellow-colored zone as the predator approaching region. The data are expressed as the median with interquartile range and were analyzed by a Kruskal–Wallis test, which continued with Dunn’s multiple comparisons test as a follow-up test (*n* = 30 for control; *n* = 19 for 0.5 ppm PS-NPs-exposure fish; *n* = 20 for 1.5 ppm MP-exposure fish; *p* < 0.05).

Fear is a collection of behavioral responses that are elicited by negative stimuli that are associated with imminent danger, such as the presence of a predator. Predator avoidance is an innate response for fish when facing their natural predator by showing high anxiety or even freezing behavior. Zebrafish have an innate response as freezing or anxiety when exposed to the sight of a natural predator. Therefore, this response is helpful in examining certain behavior alterations during the course of revelation to the predator [28]. In the predator avoidance test, we assessed zebrafish fear reactions, including predator avoidance behavior. We exposed the different groups of zebrafish to the predator fish convict cichlid (*Amatitlania nigrofasciata*). From the results, we found that exposure of PS-NPs at 0.5 ppm did not alter zebrafish fear response to the predator, as shown in Figure 4. No significant difference was detected in all of the endpoints measured during the test between control and 0.5 ppm PS-NPs-exposed fish group (Figure 4A–H). However, exposure of PS-NPs at a higher concentration (1.5 ppm) did alter their fear response to the convict cichlid. This difference was indicated by the low level of the average distance to separator between zebrafish and predator fish exhibited by high concentration PS-NPs-exposed fish (Figure 4B). In addition, there was also a slight increment in their predator approaching time, even though it did not reach a statistically significant difference (Figure 4A). Furthermore, we found a difference in 1.5 ppm PS-NPs-exposed fish movement types with the control fish, even though there was no significant difference in the average speed (Figure 4C). This difference was indicated by a high level of swimming time movement ratio, while there were no significant differences in their freezing and rapid movement time ratios (Figure 4D–F). These results suggest that 1.5 ppm of PS-NPs exposure reduces the predator avoidance behavior performance in zebrafish. In addition, Figure 4G–I and Supplementary Video S3 illustrate the locomotion trajectories and behavioral changes for the control, 0.5 and 1.5 ppm PS-NPs-exposed fish in the predator avoidance test.

### 2.4. PS-NPs Exposure Did Not Alter Conspecific Social Behavior in Zebrafish

**Figure 5:**
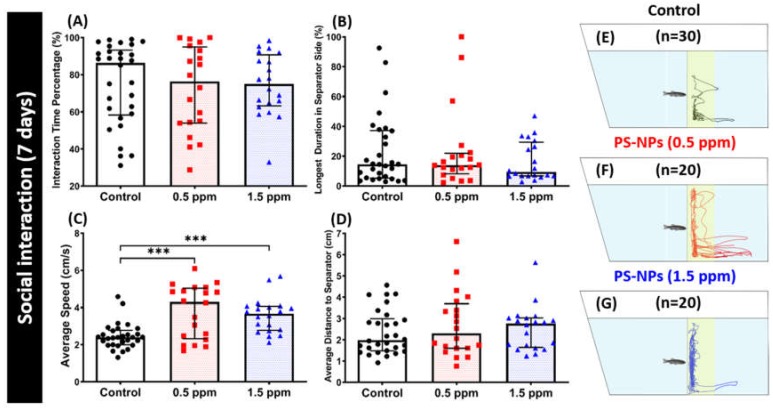
Social interaction behavior endpoints comparisons between control and polystyrene nanoplastics (PS-NPs)-exposed zebrafish groups after a ~7-day exposure. (**A**) Interaction time percentage, (**B**) longest duration in the separator, (**C**) average speed, and (**D**) average distance to separator side were analyzed for social interaction assay. The 1 min. locomotion trajectories for the control, 0.5 and 1.5 ppm PS-NPs exposed fish in mirror biting tests were presented in **E**, **F**, **G**, respectively with the yellow-colored zone as the conspecific interaction region. The data are expressed as the median with interquartile range and were analyzed by a Kruskal-Wallis test, which continued with Dunn’s multiple comparisons test as a follow-up test (*n* = 30 for control; *n* = 20 for each PS-NPs-exposed group).

We assessed zebrafish social nature by conspecific interactions by performing social interaction tests. We tested their social interest to another conspecific after a one-week exposure of PS-NPs. Here, we did not find any significant change between the experimental and untreated groups in terms of interaction time percentage (Figure 5A), longest duration in separator side (Figure 5B), and average distance to the separator (Figure 5D). However, the average speed was increased in the experimental groups when compared to untreated controls (Figure 5C), which is consistent with the novel tank test result. This result suggests that both 0.5 and 1.5 ppm of PS-NPs exposure does not change the conspecific interaction in zebrafish. Figure 5E–G and Supplementary Video S4 illustrate the locomotion trajectories and behavioral changes for the control, 0.5 and 1.5 ppm PS-NPs-exposed fish in the social interaction test.

### 2.5. PS-NPs Exposure Tighten the Shoal in Zebrafish

**Figure 6:**
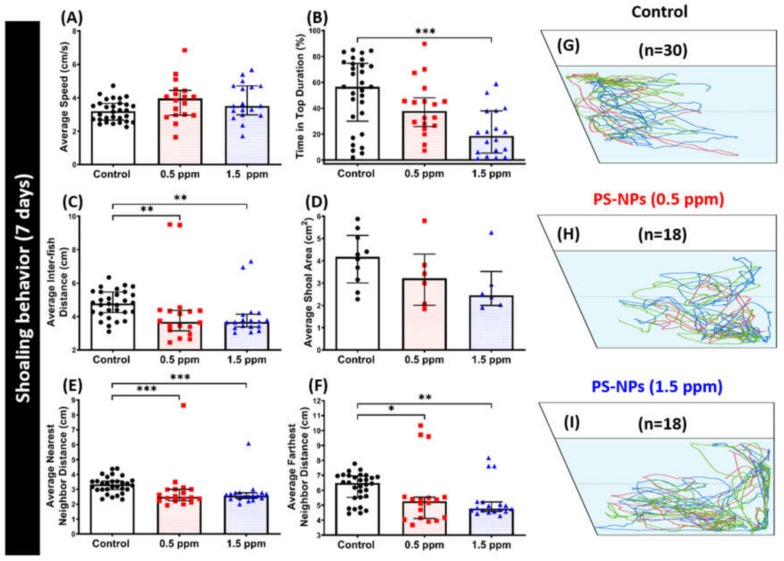
Shoaling behavior endpoint comparisons between the control and polystyrene nanoplastics (PS-NPs)-exposed zebrafish groups after a ~7-day exposure. (**A**) Average speed, (**B**) time in top duration, (**C**) average inter-fish distance, (**D**) average shoal area, (**E**) average nearest neighbor distance, and (F) average farthest neighbor distance, were analyzed. Groups of three fish were tested for shoaling behavior. The 1 min. locomotion trajectories for the control, 0.5 and 1.5 ppm PS-NPs exposed fish in shoaling tests were presented in **G**, **H**, **I**, respectively. The data are expressed as the median with interquartile range were analyzed by a Kruskal–Wallis test, which continued with Dunn’s multiple comparisons test as a follow-up test (*n* = 30 for control; *n* = 18 for PS-NP-exposed fish; * *p* < 0.05, ** *p* < 0.01, *** *p* < 0.005).

Shoaling is an inherently social behavior of zebrafish. Shoaling is an innate behavior for fish to swim together to reduce anxiety and the risk of being captured by predators. When zebrafish sense a certain kind of threat or when challenged in a situation they tend to avoid, they usually swim into very tight groups together. Thus, the shoaling test can specify certain neurological behaviors of zebrafish after PS-NPs exposure. Shoaling nature provides the individual fish with multiple benefits, including efficient foraging, defense against predators, and access to mates [29,30]. From the result, we found tight shoal were formed by the PS-NPs-treated fish. This phenomenon was supported by a decrease in average inter fish distance, average nearest neighbor area, and average farthest neighbor distance showed in both the 0.5 ppm and 1.5 ppm PS-NPs-treated groups (Figure 6C,E,F). In addition, there was a slight decrement in their average shoal area, even though it did not reach a statistically significant difference (Figure 6D). Furthermore, 1.5 ppm of PS-NPs exposure reduced fish group exploratory behavior, which was indicated by a decrease in time spent at the top portion of the tank (Figure 6B). However, there was no significant difference regarding their locomotion activity observed during this test (Figure 6A). This result suggests both 0.5 and 1.5 ppm of PS-NPs exposure trigger a tight shoaling behavior in zebrafish. Figure 5G–I and Supplementary Video S5 illustrate the locomotion trajectories and behavioral changes for the control, 0.5 and 1.5 ppm PS-NPs-exposed fish in the shoaling test.

### 2.6. High Dose of PS-NPs Exposure Dysregulated the Circadian Rhythm

**Figure 7:**
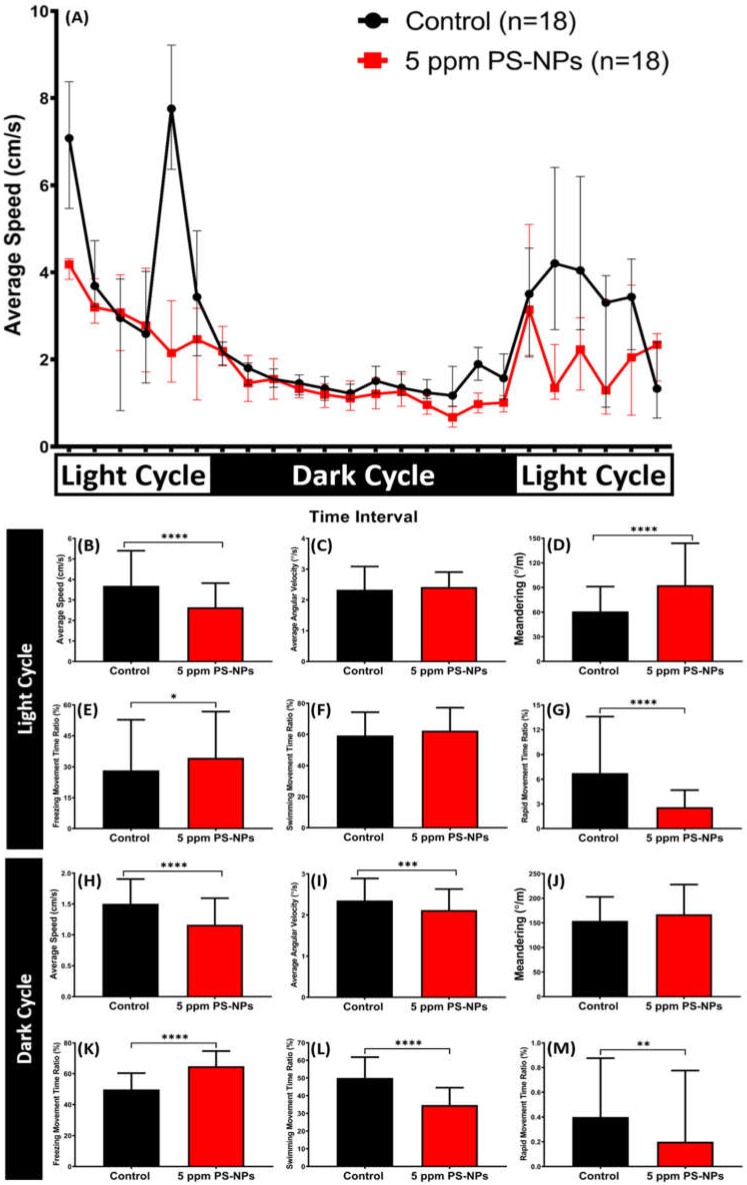
The circadian rhythm locomotion activity assay for control and 5 ppm polystyrene nanoplastics (PS-NPs)-exposed zebrafish groups after a seven-week exposure. (**A**) Comparison of the time chronology changes of the average speed between the control and the PS-NPs-exposed fish in light and dark cycles. The white area shows the light period and the black area shows the dark period. Comparison of the average speed (**B**,**H**), average angular velocity (**C**,**I**), and meandering (**D**,**J**), freezing movement time ratio (**E**,**K**), swimming movement time ratio (**F**,**L**), and rapid movement ratio (**G**,**M**) during the light and dark cycles, respectively. The data are expressed as the median with interquartile range and were analyzed by Mann–Whitney test (*n* = 18 for control; *n* = 18 for PS-NPs-exposed fish; * *p* < 0.05, ** *p* < 0.01, *** *p* < 0.001, ****, *p* < 0.0001).

Circadian rhythm genes and their regulation are well documented, particularly in the zebrafish brain and in pineal independently in several vertebrates [31,32]. In this test, we assessed zebrafish circadian rhythm locomotion activity after a high dose of PS-NPs was exposed chronically. The result showed a chronic exposure of 5 ppm PS-NPs dysregulated their circadian rhythm locomotion activity (Figure 7A). In the light cycle, the reduction of locomotion activity and abnormal movement orientation were observed in the treated fish, which was indicated by the significant reduction in average speed and rapid movement time ratio of PS-NPs-exposed fishes (Figure 7B,G), followed by the increment in meandering and freezing movement time ratio (Figure 7D,E). However, there were no significant differences found in the average angular velocity and swimming movement time ratio between the control and treated groups (Figure 7C,F). Furthermore, a similar phenomenon was also shown during the dark cycle. In the dark cycle, more robust hypoactivity behavior was exhibited by the treated fish, supported by the reduction of average speed, average angular velocity, swimming movement ratio, and rapid movement ratio of the PS-NPs-treated fish as compared to the controls (Figure 7H–I,L–M). In addition, the high level of freezing movement time ratio might also support this behavior alteration (Figure 7K). However, there was no statistical difference in their meandering during the dark cycle (Figure 7J). This result suggests that the chronic exposure of 5 ppm PS-NPs dysregulates circadian rhythm locomotion activity in zebrafish.

### 2.7. Tissue Distribution of Fluorescent PS-NPs in Zebrafish

**Figure 8:**
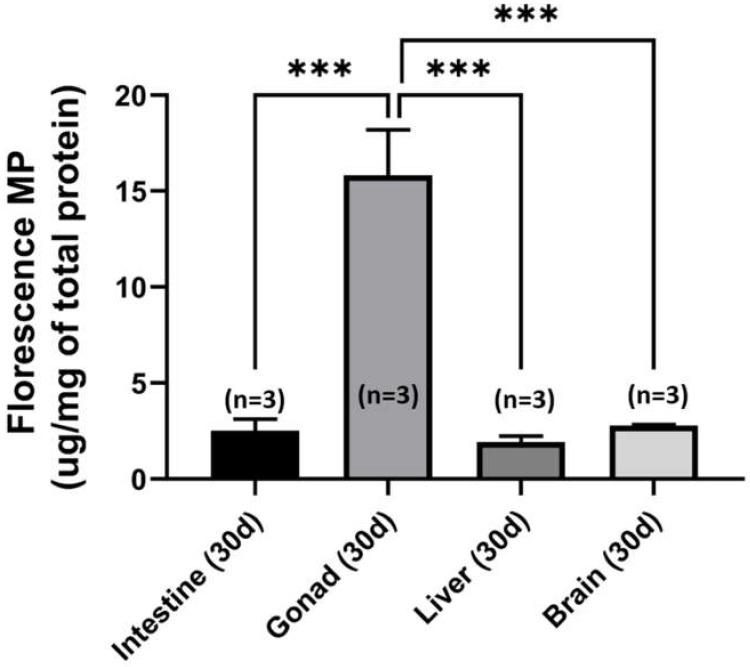
Comparison of the tissue distribution of green fluorescence-labeled nanoplastics among different tissues in zebrafish after ~30 days of polystyrene nanoplastics (PS-NPs) exposure. The data are expressed as the mean ± SEM and they were analyzed by One-way ANOVA, which continued with post hoc analysis (*n* = 3; *** *p* < 0.001).

We performed fluorescence spectroscopy analysis on the treated tissues, which were collected from the emission spectra 489 nm to 590 nm, at an excitation wavelength of 485 nm. We found strong green fluorescence in the PS-NPs-treated tissues as compared to the controls (Figure 8). The green fluorescence was predominantly seen in gonads (can reach around 15 ug/mg total protein) and intestine, and other tissues, including liver and brains (around five times less than those in the gonads). This result demonstrates that the green-labeled PS-NPs can accumulate in various tissues, including the nervous, digestive, and reproductive organs after long term exposure.

### 2.8. Measurement of Marker Expression in Muscle and Liver after PS-NPs Exposure

Several biochemical parameters that were related to oxidative stress, energy and lipid metabolisms, oxygen uptake, DNA damage, inflammatory response, and environmental toxic responses were examined in muscle and liver tissues of the PS-NPs-exposed fish to better understand the corresponding toxic mechanism induced by PS-NPs (summarized in Table 1). From the muscle tissues, we examined oxidative stress, energy metabolism, and oxygen uptake changes after PS-NPs exposure. Regarding oxidative stress, we found that the relative amount of reactive oxygen species (ROS) level increased in the fish exposed to the high concentration of PS-NPs while we found that a high concentration of PS-NPs induced a significant decrease in ATP level for energy metabolism. On another hand, a significant change was absent in both treated groups regarding their level of creatine kinase, a muscle energy marker. Furthermore, from this tissue, we also found that Hif-1α, a key marker for hypoxia, was not changed after the exposure.

Next, the effects of PS-NPs exposure on DNA damage, inflammation, and lipid peroxidation were evaluated by measuring several important biomarkers, such as single-stranded DNA (ssDNA), TNF-α, and malondialdehyde (MDA) from the liver tissue. Regarding the DNA damage, the relative amount of ssDNA was increased in the high concentration group. Furthermore, in the 0.5 ppm PS-NPs-treated group, the relative amount of TNF-α, an inflammatory marker, was not significantly different from those of the control. However, the relative amount of those markers in the liver of 1.5 ppm PS-NPs-treated group were significantly higher than those of the control. In addition, MDA, which is a marker for lipid peroxidation, the relative amount from the liver tissue was unchanged in both groups. Several biomarkers for chemical exposure-response were also measured from the liver tissues, including cortisol and ethoxyresorufin-O-deethylase (EROD). From the result, we found a significant increment in the cortisol level exhibited by the high concentration group only. However, there were no significant differences regarding the EROD level that was observed in all of the groups. In addition, we also found increments in the vitellogenin (VTG), a biomarker for environmental estrogens, levels in both treated groups. Later, we analyzed the regulation of three isoforms of cytochrome P450, CYP enzymes (CYP1A1, CYP11A1, and CYP19A1) in zebrafish liver after the PS-NPs exposure. Interestingly, all three CYP isoenzyme expressions were significantly elevated in 1.5 ppm PS-NPs-exposed zebrafish when compared to the control.

### 2.9. Measurement of Neurotransmitter Expression in Brain after PS-NPs Exposure

Neurotransmitters affect a wide variety of both physical and psychological functions, including heart rate, sleep, appetite, and behaviors, such as mood and fear. The expression of neurotransmitters in the brain was measured biochemically while using enzyme-linked immunosorbent assay (ELISA) to investigate PS-NPs exposure to neurotransmitters. Fixed amount of the total soluble protein in brain was subjected to ELISA to determine the expression level of neurotransmitters, such as acetylcholine esterase (AChE), acetylcholine (ACh), dopamine (DA), melatonin, γ-aminobutyric acid (GABA), serotonin (5-HT), vasopressin, kisspeptin, prolactin (PRL), oxytocin, and vasotocin. From the result, the activity of AChE was significantly inhibited in the 1.5 ppm PS-NPs group (Table 1). Interestingly, the relative amount of ACh did not significantly change in both of the treated groups. Furthermore, the other neurotransmitters, such as DA, melatonin, GABA, 5-HT, vasopressin, kisspeptin, and oxytocin, were also significantly decreased after exposure to PS-NPs, especially in the high concentration group. However, the relative amount of PRL and vasotocin did not show any appreciable change in the treatment groups.

## 3. Discussion

### 3.1. Microplastic Pollutions

Over the past few years, very high concentrations of microplastics have been detected in freshwater bodies (0–1×10^6^ items/m^3^) [33] and oceans (0–1×10^4^ items/m^3^) [34]. It was calculated that between 4.8 and 12.7 million metric tons of plastic waste disposed into the ocean in 2010 and this mass could drastically increase by one order of magnitude by 2025 [1]. Microplastics enter the ecosystem from many sources, including clothing, industrial processes in the form of microbeads, plastic pellets, and microfibers that degraded from plastic bags and fishing nets. Plastics take hundreds to thousands of years for degradation, thus increasing their probability of being ingested and accumulated in bodies and tissues of water organisms. The evidence of this incorporation of microplastics in the organism bodies has already been observed in various species of animals, particularly fishes and mussels that are commonly used for human consumption. It is becoming increasingly evident that microplastics can be transmitted through the aquatic food web, which leads to biological accumulation. The entire movement and life cycle of microplastics in the environment is still under research, in a critical review and assessment of data quality of the occurrence studies of microplastics, Dr. Koelmans and colleagues at the Wageningen University, the Netherlands assessed the quality of fifty studies researching microplastics in drinking water and its major freshwater sources. They concluded that more high-quality data are needed on the occurrence of microplastics in drinking water, to better understand potential exposure and inform human health risk assessments [35]. Additionally, in a study in *Mytilus edulis*, the accumulation of microplastics in the form of microfibers varied from 0.9 to 4.6 items/g, where more microplastics were detected in the wild groups than in the farmer’s group [36]. In the current study, we evaluated the effects of several different concentrations of PS-NPs on the adult zebrafish behavior and physiological aspects with an environmentally relevant concentration after chronic incubation. The obtained results added new important information regarding the neurotoxic effects of this emerging pollutant on adult zebrafish.

### 3.2. Inflammatory Protein Expression and Oxidative Stress

We investigated the inflammatory protein expression and measured oxidative stress to understand the exacerbation of PS-NPs induced toxicity in zebrafish. First, we evaluated the level of TNF-α marker as a representative protein of inflammations by ELISA. The 1.5 ppm treatment induced the marker level to rise in liver tissues and triggered a significant immune response, thus suggesting a synergistic effect on inflammation. Furthermore, the lack of anti-inflammatory function observed from liver tissue when fish were exposed to a high concentration of PS-NPs with elevated ssDNA, suggesting an oxidative stress mechanism of toxicity [37]. Reactive oxygen species (ROS) are one of the important features that result from toxicant-induced cell death and they are implicated in the inflammatory response. ROS generation by PS-NPs was examined using the ROS ELISA kit showing ROS production was dramatically increased in the PS-NPs 1.5 ppm treated group, indicating that the presence of PS-NPs synergistically aggravated ROS production. In a prior study, when 0.05, 0.5, & 6 µm polystyrene beads were exposed to rotifers, different sizes of microbeads induced ROS in a size-dependent manner [38]. Besides, the size dependency of microbeads side effects was proved as a consequence of oxidative stress, which is P-JNK, P-p38 activation with increased ROS level [38]. Smaller sized PS-NPs had been shown to induce the activities of ROS and Hif-1α biomarkers. These studies demonstrated that smaller sized particles are more toxic than the larger particles due to their specific surface area [39,40]. Moreover, a high level of ROS might also be due to insufficient nutrition or the inhibition of fish food digestion that is caused by aggregated PS-NPs in fish to a larger extent, although they were not aggregated in the water. In addition, even though the ROS levels were investigated after ~7 days of PS-NPs treatment, the aggregation and accumulation of fluorescence-labeled PS-NPs in fish determined after ~7 weeks of accumulation may also be related to this phenomenon. More future experiments are considered to be conducted to validate this hypothesis. Additionally, polyethylene and polystyrene microplastics were shown to adsorb pyrene in time and dose-dependent manner in *Mytilus galloprovincialis*, depicting cellular deformities with an alteration in oxidative stress, neurotoxic effect, and antioxidant system in a previous study [18]. Therefore, this result might be an important consideration for future research efforts. To conclude, the high concentration and size of nanoplastics appeared to be one of the key determinants of such toxicity, leading to elevated ROS production and cell death, as well as pro-inflammatory responses.

### 3.3. Energy Metabolism

The toxic effects of PS-NPs in the liver of zebrafish were investigated after ~7 days of exposure. In our study, the altered level of ATP marker in a biochemical assay confirmed the disruption of energy metabolism. Similar studies have reported that in marine worms and copepods, ingested NPs depleted energy reserves [24,41] and particularly affected the feeding behavior of fish [42]. A possible explanation is that the large quantity of ingested PS-NPs without any nutritional value hindered the normal absorption of food. In addition, the dysregulation of food intake can also lead to severe changes in energy and lipid metabolism.

### 3.4. Reproductive Toxicity of PS-NPs

In a previous study, the 2 and 6 µm diameter and 0.023 mg/L of micro-PS significantly decreased the oocyte number, diameter, and sperm velocity in oysters when exposed for two months, after assessing the reproductive cycle on different eco-physiological parameters [43]. Based on the biochemical analysis of differentially expressed CYP proteins, PS-NPs may directly affect the ovary. Moreover, nanoplastics may cause oxidative stress to the developing follicles and interfere with the expression of key regulatory genes, impairing the function and growth of stage 1/11 follicles. The aborted follicular development, as well as the inhibition of E2 synthesis, could be a consequence of cyp1a1 down-regulation [44,45,46]. Generally, the accumulation of yolk in oocytes during oocyte development after fertilization is a key success in zebrafish embryonic development [47]. Therefore, vitellogenin (VTG), a female-specific protein that is responsible as a precursor of egg yolk proteins in vertebrates, plays an important role in zebrafish reproduction [48]. This protein is synthesized in the liver, secreted to the bloodstream, and then transported to the developing oocytes for vitellogenesis process [49]. Thus, VTG has been used as a biomarker of estrogenic pollution or as a biomarker of an endocrine disruptor in vertebrates [50,51,52]. In addition, male zebrafish also can synthesize and secrete VTG when exposed to estrogen mimic pollutants, even though VTG is a female-specific protein [53,54]. In this experiment, the increased level of VTG in the treated fish suggests that PS-NPs caused endocrine disruption pollution in the environment.

Later, a fluorescence spectrophotometer was used to quantify fluorescence coated PS-NPs that accumulated in tissues of zebrafish after one-month incubation. Even though this technique relies on the fluorescent dye encapsulated in plastic beads, it is very useful to quantify the accumulation of PS-NPs in different tissues after the exposure. It is noted that, in zebrafish, the gills are considered as the initial site for uptake and elimination of nanoparticles, while the brain, gonads, and liver connected with the gills via arterial blood. In addition, blood circulating in veins from the gonads will also reach the liver [55]. We found that 70 nm PS-NPs in low concentration preferentially accumulated in the gonad as compared to other tissues/organs, in accordance with previous studies in another nanoparticle [56]. This result indicated that PS-NPs could pass through the gonad blood barriers and accumulate in gonad tissues. These gonadal alterations would possibly further affect reproductive activity by inducing germ cells apoptosis in gonad tissues [57]. In another prior study, this issue was shown in the zebrafish when their parents were exposed to the poly-N-vynil-2-pirrolidone and polyethylenimine (PVP/PEI) coated silver-nanoparticles. Parental exposure to Ag-NPs increased embryo malformation prevalence, suggesting that silver is able to reach the gonads and accumulate in the oocytes causing a disturbance in developing embryos in fish waterborne exposed to the Ag-NPs suspension [58]. Furthermore, nanoparticles were also found to invade the protective barrier and disrupt the oocytes in the mice study. In their study, NPs crossed the blood-brain barrier and accumulated in the central nervous system (CNS) [59]. Later, NPs disrupted hormone secretion, such as gonadotropin-releasing hormone (GnRH), which is responsible for oogenesis, through the hypothalamic-pituitary-gonadal axis [60,61,62]. Moreover, NPs could also cross through the placenta into the fetus, causing treated mice to likely exhibit fetal inflammation, genotoxicity, apoptosis, reproductive deficiency, and immunodeficiency [60]. In addition, another study in *Bombyx mori*, an invertebrate model organism, also proved that Ag-NPs could induce reproductive toxicity. In this study, the possible mechanism is Ag-NPs passed through the gonads and generated ROS in spermatocytes and internal germ cells, inducing the early germ cell death that led to the cells and DNA damage. The genetic integrity of the gonads is an essential aspect of reproductive success [63]. Thus, as ROS can harm the DNA and cells, this damaged genetic will be inherited to the next generation as the defective genetic [64,65]. In summary, even though the direct evidence that the PS-NPs passed through the bio-barrier and moved to further tissues in fish, such as gonads and brain, could not be shown; the fact that these organs were affected become an indicator from the perspective of biological effects. The alterations that were observed in several tissues demonstrate that PS-NPs do exert potential toxic effects on both the reproductive and nervous systems.

### 3.5. Behavioral and Neurotransmitters Alterations Caused by PS-NPs

The novel tank test has emerged as a potentially useful behavioral measure of anxiety in zebrafish. It exploits the tendency of zebrafish to initially dive to the bottom of a novel experimental tank, which has been compared to thigmotaxis in rodents, with a gradual increase in vertical activity over time [25]. Similar to the rodent open-field test, the novel tank test endpoints can be applied to zebrafish models of anxiety [66]. During the novel tank test, similar locomotion activity and exploratory behavior between control and low concentration of PS-NPs-exposed groups were observed. On another hand, the high concentration of the PS-NPs-exposed group exhibited hyperactivity and abnormal exploratory behaviors. Various important neurotransmitters in the brain were measured by performing ELISA with antigen-specific antibodies to understand the molecular mechanisms involved in this behavioral impairment of PS-NPs exposed to zebrafish. Abnormal behavior showed by the treated group might be associated with the deregulation of several important biomarkers, including oxytocin, vasopressin, and kisspeptin. Oxytocin, which is known to buffer the stress response, in the brain modulates a broad variety of behaviors, as well as anxiety-related behavior and stress coping. Together with vasopressin, oxytocin is an essential part of the hypothalamo-neurohypophysial system that appears to activate bond-relevant behaviors, such as the exploration of a novel environment [67,68]. In humans, oxytocin projects into the amygdala, hippocampus, and regions of the spinal cord that regulate the parasymphatic branch of the autonomic nervous system, which attenuates stress responses [69]. In addition, another study also found the antidepressant-like effects that are caused by kisspeptin-13 in mice modified forced swimming test via adrenergic and serotonergic receptors [70]. Further, recent research suggests that anxiety-like behavior is also directly associated with the acetylcholinesterase (AChE) activity of the hippocampus since AChE knockdown in the hippocampus promotes anxiety-like behavior in mice [71]. AChE is an enzyme that is responsible for the hydrolysis of the neurotransmitter acetylcholine, and it has been implicated in several non-cholinergic actions, including acute stress response and neurite outgrowth [72]. Several studies have shown that nanoplastics exposure could dysregulate AChE activity in aquatic organisms [18,73,74]. In the current study, the AChE level found to be deregulated in the treated fish that may contribute to the anxiety-like behavior that was exhibited by these fish. The previous study in mice has found that anxiety was linked to AChE activity by demonstrating that pubertal BPA (Bisphenol A) exposure increased anxiety-like behavior and decreases AChE activity in the hippocampus [75]. Another prior study also reported that exposure to high doses of thiamethoxam, a neonicotinoid insecticide, in rats produces AChE inhibition that persists some days after exposure accompanied by deficits in behavioral performance [76]. In reptiles and amphibians, exposure to AChE-inhibiting pesticides, such as carbaryl compromised their locomotion activity, which is such a critical process as a predator avoidance and prey capture [77]. Equally important, abnormal levels of several neurotransmitters, including γ-Aminobutyric acid (GABA), dopamine, and serotonin, may also play a role in this abnormal behavior. Extensive evidence indicated that GABA transmission plays a primary role in the modulation of behavioral sequelae resulting from stress, while serotonin (5-hydroxytryptamine) is implicated in the regulation of several developmental, behavioral, and physiologic processes, including anxiety and affective states in human and nonhuman species [78]. Serotonin is a key modulatory neurotransmitter in the central nervous system [79]. In humans, dysfunction in the neurotransmission of serotonin is implicated in a variety of psychiatric disorders, including major depression and anxiety [80]. As an addition, cortisol, a steroid hormone, might also contribute to this abnormal behavior. It is considered as the principal corticosteroid that is secreted by the teleost fish adrenal system in response to acute and chronic stress, which might explain the elevated level of cortisol that was observed in this study [81,82].

Aggressiveness, an ancestral behavior that is common to all animal species at least from fishes onwards, is one of the behavioral traits that is often closely linked to fitness [83]. It can be defined as the execution of action against animals belonging to the same or different species [84]. In zebrafish, mirror-image stimulation is traditionally used for studying zebrafish aggressive behavior [26]. Our findings demonstrated that both concentrations (0.5 and 1.5 ppm) of PS-NPs significantly impaired zebrafish aggressiveness in the mirror-biting test. The activity of several important biomarkers was also evaluated to investigate the mechanism related to this behavior alteration. From the result, significant reduced oxytocin and vasopressin activities observed in the brain tissue may be related to the aggressiveness reduction. Supporting the current result, previous studies showed that oxytocin increased the sexual and aggressive behavior of dominant monkeys and modulated a broad variety of behaviors, including pair bonding and aggression in rats [68,85]. Furthermore, aggressive behavior is partially influenced by vasopressin, a neuropeptide hormone implicated in the regulation of several social behaviors, mostly through its receptors in the brain [69,86,87]. In mice, a lower binding of vasopressin to its receptor 1a is connected with lower aggression, and knocking out the vasopressin receptor 1b significantly reduces its aggressive behavior. In addition, the injection of arginine vasopressin into the ventrolateral hypothalamus was found to stimulate aggressive behavior in gonadally intact male mice [88]. Generally, in mammals, oxytocin and vasopressin neurons are both involved in the control of social behaviors, such as aggression and reproduction [89,90]. The dysregulation of several important brain neurotransmitters, including dopamine and serotonin, might also be related to the less aggressive behavior observed in treated fish since these transmitters are critically involved in the neural circuits for many types of human and animal aggression [91]. In preclinical studies, the role of dopamine D1, D2, and D3 receptors in the modulation of aggression has been documented. Moreover, more persuasive evidence for a significant role of dopamine D2 receptors was mentioned from studies that focus on defensive-aggressive behavior in cats [92]. Traditionally, several studies have shown that elevated serotonin and GABA levels lead to decreased aggression in many different species, including humans [92]. However, the aggression level decreased in the treated fish along with downregulated serotonin activities in the present study. One possible explanation is that the majority (95%) of total body serotonin is released into the gut by intestinal enterochromaffin cells, which might compensate for the downregulated serotonin in the brain that is caused by the microplastic NP exposure [93].

As one of the important behavioral reactions, fear responses may have a significant fitness component, since it might allow the animal (and human) to avoid predation or other forms of danger in nature [94]. Based on the previous method, fear responses were induced in zebrafish by presenting a convict cichlid (*Amatitlania nigrofasciata*) during the predator avoidance test [95]. In the present study, alteration of predator avoidance behavior was shown by the high concentration of the PS-NPs-treated group, while this phenomenon was absent in the low concentration of the PS-NPs-treated group. Later, we quantitatively compared the neurotransmitters and other biochemical markers between the control and PS-NPs-treated fish. From the result, we found a significant decrease in kisspeptin levels after the PS-NPs treatments to the zebrafish. Kisspeptin is a hypothalamic neuropeptide that is derived from the *Kiss1* and it has proven to play a major role in vertebrate reproduction. However, several studies demonstrated a unique role for the kisspeptin system in altering the fear response in several animal models. In mice, the effects of kisspeptin-13 on mice passive avoidance learning were reported. Kisspeptin-Kiss-R signaling could be involved in the contextualization of fear in mammals since the expression of the *Kiss1* gene and Kiss-R was shown in the hypothalamus and the medial amygdala, which is a fear-regulating region in rodent brain [96]. In addition, very recent findings showed a unique role of kisspeptin in inhibiting fear response in zebrafish. Thus, it suggests the interaction between the vHb-expressing *Kiss1* and the serotonin system in the modulation of alarm substance-evoked fear responses [70]. Furthermore, the decreased activities of vasopressin and oxytocin may also related to this behavioral impairment, because these hormones are well known as modulators of a variety of cognitive and emotional processes, most notably learning and memory, trust and selective affiliation, and fear [90]. A pioneering study also found that oxytocin reduced amygdala activation and its coupling to brainstem centers responsible for autonomic and behavioral components of fear [69]. The low level of GABA was also observed in the treated fish brain. GABA is the most abundant inhibitory neurotransmitter in the mammalian brain and is known to be related to the anxiety/fear response in human and animal models [97,98]. In the rat study, it has been found that GABA neurotransmission in the nucleus accumbens shell played a role in defensive or fear-related behavior, which might contribute to the diminished predator avoidance behavior exhibited by the treated fish during the assay [99].

Social interactions are an important domain of animal and human behavior since they play a major role in the acquisition and development of learned behaviors [100]. Social interaction and shoaling behavior tests are very commonly used behavior assessments in fish models to study their social behaviors. In zebrafish, these tests assess their sociability by observing the interactions between several fishes [26]. Even though social behavior deficit was not shown by the PS-NPs-treated fish during the social interaction test, tighten shoals were formed by both of the treated groups, which might indicate stress responses in zebrafish [101]. We found that the reduction of oxytocin and vasopressin levels might play roles in this phenomenon after several major brain neurotransmitters were measured. As mentioned earlier, oxytocin and vasopressin are both best known for their contribution to the regulation of social behavior; moreover, a plethora of studies in humans already found that both of these neuropeptides modulate human social behavior and cognition [68,102]. In the previous study, the oxytocin effect in the social behavior modulation was studied in the oxytocin knockout mice. The mutant mice failed to recognize familiar conspecifics after repeated social encounters while it can be restored with central oxytocin administration into the amygdala [103]. Thus, the effects of oxytocin on partner preferences strongly suggest a causal connection between oxytocin and the formation of social bonds [67]. Furthermore, vasopressin was found to modulate social behaviors and motor activity in both dominant and subordinate monkeys [85]. Finally, comparisons across fishes, amphibians, and mammals also indicate that oxytocin and vasopressin and their homologs both regulate many of the same types of social behaviors throughout the vertebrate lineage [90]. In addition, the development of shoaling has also been found to be associated with whole-brain GABA and dopamine levels [104]. Previously, a study demonstrated that, in response to social stimuli, dopamine and 3,4-Dihydroxyphenylacetic acid (DOPAC) levels rapidly rise in the brain of adult zebrafish [105]. Dopamine D1-receptor antagonism was also found to impair shoaling in adult zebrafish in a dose-dependent manner [106]. In goldfish, the tendency to shoal has been demonstrated to increase when they were administered with anxiogenic drugs-GABA agonists [107]. Further, the study in medaka (*Oryzias latipes*) was also shown that an anti-anxiety drug—the positive allosteric modulator of GABA diazepam—selectively modified their shoaling behavior [98]. Taken together, the low level of dopamine and GABA that was observed in this research might contribute to the abnormal shoal that formed by the PS-NPs-treated fish group.

Circadian rhythms play a central role in adapting the physiology and behavior of living organisms to anticipate daily environmental changes [108]. After seven weeks of PS-NPs exposure, the dysregulation of circadian rhythm locomotion activity was shown by the treated fish group and might be related to the melatonin, a key hormone controlling the circadian rhythm. Generally, this physiological hormone gets involved in sleep timing and currently used as a primary treatment for sleep disorders in humans [109]. Melatonin has also been reported to promote sleep-like behavior in diurnal vertebrates, such as zebrafish, and is responsible for other physiological processes, including immune function, blood pressure, and retinal physiology [110,111]. However, in this experiment, PS-NPs-treated groups both showed a significantly low level of melatonin, while sleep-like behaviors were observed during the circadian rhythm locomotion activity test in light and dark cycles. One possible explanation is that the sleep-like behavior showed by the treated fish was likely caused by anxiety or depression, not by the melatonin level [112]. The downregulated vasopressin level might also contribute to this behavior alteration since vasopressin is the product of one such clock-controlled gene that evokes circadian rhythms. The previous study reported that the circadian rhythmicity of locomotor activities was significantly reduced in V1a (one of the vasopressin receptor)-deficient mice [113]. In addition, there is a possibility that serotonin also plays a role in this phenomenon. This is supported by another prior study in mice lacking the serotonin transporter. The abnormality in REM sleep was shown by the mutant mice, followed with other abnormalities, including reward-related and locomotor responses to psychostimulants and analgesic responses [114]. Besides, the hypoactivity behavior of fish, in this case, might also be attributed to an increase in oxidative damage [2]. However, this hypoactivity behavior that was observed during this test was in contrast with the hyperactivity behavior shown during the novel tank test. A possible explanation is that higher concentration (5 ppm) and longer exposure of PS-NPs were applied in the circadian rhythm locomotion activity test, while, in the novel tank test, only lower concentrations (0.5 and 1.5 ppm) and shorter exposures of microplastic PS-NPs were used.

Taken together, we found that PS-NPs treatments inhibited the activity of several important neurotransmitters at the highest concentration of PS-NPs, which might lead to cholinergic neurotransmission insufficiency. These dysregulations raise the potentiality that PS-NPs exposure could induce adverse effects on neurotransmission in zebrafish. This investigation is a step in the direction of understanding the adverse effect of PS-NPs on freshwater or marine organisms to understand the phenomenon at a very basic level.

## 4. Conclusions

**Figure 9:**
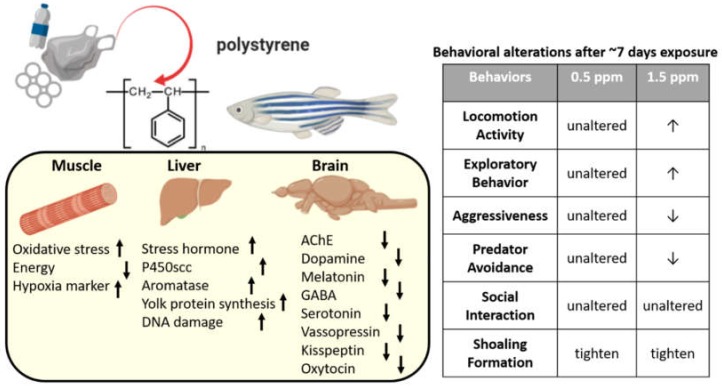
Schematic diagram of the biochemical and behavioral changes after polystyrene nanoplastics (PS-NPs) exposure in zebrafish. Left panel showing the biomarker expression alteration (↑: up regulated, ↓: down regulated) in the muscle, liver, and brain after PS-NPs exposure. Right panel showing the behavioral alteration after PS-NPs exposure at either 0.5 or 1.5 ppm (↑: higher behavior level, ↓: lower behavior level).

To the best of our knowledge, the behavior impairments that result from the exposure to 70 nm PS-NPs have not been investigated before in fish. Our study confirmed that acute (~7 days) and chronic (~7 weeks) exposure of 70 nm PS-NPs could alter the neurobehavior and accumulated in at least four tissues (brain, liver, gonads, and intestine). Moreover, PS-NPs accumulation induced several effects on behavioral profiles and biochemical biomarkers predicting the potential health risk to mammals (as summarized in Figure 9). We provide evidence that PS-NPs exposure causes disruptions to behavior, induces oxidative stress, and elicits neurotoxic responses based on a comprehensive analysis of multiple parameters. Further studies are needed to better understand the mechanism underlying the biological effects of PS-NPs on the aquatic biosystem. Other factors, such as shape, size, and composition of PS-NPs, exposure period, and physiological characteristics of the exposed organisms may be closely related to the toxic effects of PS-NPs and interlacing PS-NPs and aquatic biota.

## 5. Materials and Methods

### 5.1. Particle Characterization

Polystyrene (PS) particles (nominal size ~70 nm) were purchased from Tianjin BaseLine ChromTech Research Centre (Tianjin, China). According to the manufacturer, the particles are spherical, white opaque, and can be detected by Transmission Electron Microscopy. The PS-NPs solutions were prepared with purified water (Milli-Q, Darmstadt, Germany) and sonicated before every usage. Lumisphere uniform green fluorescent polystyrene microspheres (~70 nm) were purchased from Tianjin BaseLine ChromTech Research Centre (Tianjin, China) to study the tissue distribution after waterborne exposing to zebrafish. The manufacturer performed transmission electron microscopy to verify the particle size.

### 5.2. Zebrafish Husbandry

Healthy adult zebrafish (~6 months old and 0.30 ± 0.022 g in wet weight) were maintained at 25 ± 1 °C with a 14/10 h light/dark cycles in culture water (UV sterilized and well-aerated water, pH 7.2 + 0.4, dissolved oxygen, 6.5 ± 0.2 mg/L, electrical conductivity, 0.254 ± 0.004 mS/cm, water hardness, 183 ± 5 mg of CaCO_3_/L). The Committee for Animal Experimentation of the Chung Yuan Christian University approved all of the experimental protocols and procedures involving zebrafish (Number: CYCU107030, issue date 19 Dec. 2018). All of the experiments were performed in accordance with the guidelines for laboratory animals.

### 5.3. Exposure of PS-NPs

Adult zebrafish were randomly placed into three different glass tanks, each tank containing 20 fishes and the 2 L test solutions. During the entire experiment period, the glass tanks were continuously aerated to maintain the complete dispersion of the particles in the test solution and make sure that no aggregation was observed. For the toxicity test, fishes were randomly assigned to control and PS-NPs treated group (*n* = 20 for each group). For the treatment groups, 70 nm virgin PS-NPs were used and the exposure concentrations were 0.5 ppm, 1.5 ppm, and 5 ppm, which were chosen in accordance with the previous study about the toxicity of NPs on aquatic organisms [38]. The exposure protocol includes acute (~7 days) and chronic (~30 days and ~7 weeks) validation for the PS-NPs toxicity. For the experiment, the test solution in each tank was refreshed every two days.

### 5.4. Adult Behavior Test Battery

8 ± 1 days exposed adult zebrafish were tested mostly within the morning until afternoon (10.00 to 16:00) and start with a ~3–5 min. pre-acclimation in the tank, excluding the novel tank test, in a series of five behavioral assays: novel tank, mirror biting, predator avoidance, social interaction, and shoaling tests, as referenced from the previous publication [95]. The whole experiment was conducted in a temperature-controlled room (25 ± 1 °C). Novel tank and shoaling tests were performed on the seven days exposure, while mirror biting and social interaction tests were carried out on the following day. Lastly, a predator avoidance test was conducted on the nine days of exposure. Every day, the experiment began after the routine morning brine shrimp feeding. Fish tanks designated for conducting the experiment were transferred to the behavior testing room and freshly made system water was used in all of the testing apparatus. In these assays, untreated adult zebrafish and PS-NPs-exposed fishes were placed in an experimental tank filled with ~1.25 L of fish water. Canon EOS 600D camera with a long-range zoom lens (Canon Inc., Tokyo, Japan) was used for video recording in all of the tests mentioned above and, later, the videos were loaded by the idTracker [115] software for fish movement tracking and activity analysis based on the previous method [95,116]. In the novel tank test, the behavioral responses were recorded at a 1-min. interval of 0, 5, 10, 15, 20, 25, and 30 min. The behavioral endpoints were followed: time in top duration, average speed, freezing time movement ratio, number of entries to the top, latency to enter the top, and total distance traveled at the top. Next, the experimental tank with a mirror that was placed vertically to one side of the wall was used in the mirror biting test. After acclimation, their behavior was recorded for 5 min. The measured behavior endpoints were mirror biting time percentage, longest duration in the mirror side, average speed, freezing time movement ratio, swimming time movement ratio, and rapid movement time ratio. The tested fish were placed in the tank with a transparent glass separator placed at ~15 cm away from the vertical side of the tank wall to test the predator avoidance behavior. After acclimation, *Amatitlania nigrofasciata*, a convict cichlid, was placed on the empty side of the test tank and their behavior was recorded for 5 min. For this test, several important endpoints were calculated, including predator approaching time percentage, the average distance to the separator, average speed, freezing time movement ratio, swimming time movement ratio, and rapid movement time ratio. Later, a social interaction test was performed with a transparent glass separator that was placed at ~11 cm away from the vertical side of the tank wall. A conspecific was placed on the empty side of the test tank after acclimation. Predator approaching time percentage, average distance to separator, average speed, freezing time movement ratio, swimming time movement ratio, and rapid movement time ratio were calculated after the videos were recorded for 5 min. Finally, the shoaling assay was conducted with three fish in each test tank. After 5 min. videos were recorded, several important endpoints including average speed, time in top duration, average inter-fish distance, average shoal area, average nearest neighbor distance, and average farthest neighbor distance were calculated.

### 5.5. Circadian Rhythm Locomotion Activity Assay

A circadian rhythm locomotion activity test was performed to analyze the zebrafish locomotion activity during the light and dark cycle after ~7 weeks exposure of 5 ppm PS-NPs. This test was conducted based on a previously published method [117]. The dark/cycle test apparatus consisted of six fish tanks (20 × 10 × 5 cm), with three fishes for each tank. As a light source below the tanks, a lightbox consisting of a light-emitting diode (LED) and an infrared light-emitting diode (IR-LED) were used in light and dark cycles, respectively. One infrared-sensitive charge-coupled device (CCD; detection window: 700–1000 nm) with a maximum resolution of 1920 × 1080 pixels and a 30-fps frame rate was used for video recording (3206_1080P module, Shenzhen, China). We recorded the zebrafish locomotion activity (average speed, average angular velocity, meandering, freezing time movement ratio, swimming time movement ratio, and rapid movement time ratio) for 1 min. every hour and, later, idTracker was used to track fish movement trajectories [115].

### 5.6. Biochemical Analysis of Biomarkers

After all of the behavioral analyses, three fishes were randomly collected from each tank (nine fishes/treatment) to evaluate the toxic effects of PS-NPs, alterations of biomarkers in different tissues due to PS-NPs exposure were determined. The fish were rinsed with water to remove the microplastics from the skin. Afterward, they were anesthetized by immersing in Tricaine (MS-222) where they were sacrificed and examined later. Different tissues (muscle, liver, and brain) were collected and a pool of three zebrafish tissues was used for homogenate preparation. The tissue samples were dissected and immediately frozen in liquid nitrogen and stored at −80 °C for transcriptomic analysis and biochemical validation or fixed in 10% formalin for histopathological examinations. For transcriptomic analysis and biochemical validation, the tissues were homogenized at medium speed with a Bullet blender with 50 volumes of (*v*/*w*) ice-cold phosphate saline buffer adjusted to pH 7.2. The samples were further centrifuged at 12,000 rpm for 20 min. and the crude homogenate was stored in 100 µl aliquots at −80 °C until required. Tissues were analyzed at the end of all behavioral experiments, excluding the circadian rhythm locomotion activity, to determine the possible effects of PS-NPs exposure in the following tissues: for the muscle, oxidative stress (ROS), energy (ATP and creatine kinase), and lipid metabolisms (MDA); for liver, oxygen uptake (Hif-1α), DNA damage (ssDNA), inflammation (TNF-α), stress (cortisol), yolk protein precursor (VTG), and detoxification enzyme (EROD, CYPs); for brain, AChE, acetylcholine, dopamine, melatonin, GABA, 5-HT, vasopressin, kisspeptin, prolactin, oxytocin, and vasotocin. All of these markers were detected by using commercial ELISA kits purchased from Zgenebio Inc. (Taipei, Taiwan) and all of the measurements were conducted according to the manufacture protocols.

### 5.7. Florescence Analysis of Lumisphere Microplastics on Adult Zebrafish

Following the behavior analysis mentioned above, a one-month exposure to lumisphere microplastics on adult zebrafish was conducted for the fluorescence analysis. After ~30 days incubation with 1.5 ppm florescence PS-NPs, the fishes were anesthetized by Tricain methanesulfonate (MS222), sacrificed, and then dissected to obtain several different tissues, such as intestine, gonads, liver, and brain. Each type of tissue was used for homogenate preparation by using phosphate buffers saline. The concentrations of PS-NPs in different tissues of treated and untreated samples were measured while using a fluorescence spectrophotometer (SYNERGY-HT, Bio-Tek) with excitation 485 nm and emission at 590 nm. The standard curve was generated by using serial dilutions of lumisphere green florescence PS-NP suspensions. The background luminescence of the tissues of untreated fish was detected and then subtracted from that of the PS-NPs-exposed samples. Each detection was run in triplicate.

### 5.8. Statistical Analysis

All of the statistical analyses were performed in GraphPad Prism (GraphPad Software, Inc., version 7.01, La Jolla, CA, USA). Non-parametric analysis was used to analyze all of the behavioral and biochemical results. Post hoc comparisons were done while using Dunnett’s test unless stated otherwise. All the data are presented neither as median with interquartile range or mean ± standard error of the mean (S.E.M).

## Figures and Tables

**Table 1:** Various biomarker analyses in the muscle, liver, and brain tissues for adult zebrafish exposed to low and high doses of polystyrene nanoparticles (PS-NPs). The data are expressed as the mean ± SEM and analyzed by One-way ANOVA continued with post hoc analysis.

Biomarker	WT	PS-NPs (0.5 ppm)		PS-NPs (1.5 ppm)		Unit	Significance	ANOVA *F* Value	*p* Value
**Muscle**									
ROS	4.53 ± 0.3	5.27 ± 0.9	NS	7.31 ± 0.32	*	U/ug of total protein	YES	*F* (2, 6) =5.862	*p* = 0.0388
ATP	296.4 ± 5.80	290.3 ± 4.36	NS	242.4 ± 3.78	***	ng/ug of total protein	YES	*F* (2,6) = 39.27	*p* = 0.0004
Creatine kinase	6.70 ± 0.11	7.50 ± 0.78	NS	6.88 ± 0.39	NS	pg/ug of total protein	NO	*F* (2,6) = 0.468	*p* = 0.646
Hif-1α	18.77 ± 0.55	21.07 ± 1.6	NS	22.26 ± 0.30	NS	pg/ug of total protein	NO	*F* (2,6) = 2.974	*p* = 0.126
**Liver**									
ssDNA	2.63 ± 0.29	2.28 ± 0.61	NS	5.13 ± 0.58	*	U/ug of total protein	YES	*F* (2,6) = 9.0	*p* = 0.01
TNF-α	21.71 ± 1.67	17.59 ± 3.37	NS	45.26 ± 8.20	*	pg/ug of total protein	YES	*F* (2,6) = 8.20	*p* = 0.01
MDA	0.32 ± 0.08	0.20 ± 0.06	NS	0.27 ± 0.06	NS	pg/ug of total protein	NO	F (2,6) = 0.84	*p* = 0.47
Cortisol	64.76 ± 5.50	57.35 ± 5.50	NS	88.5 ± 6.93	*	pg/ug of total protein	YES	F (2,6) = 9.60	*p* = 0.01
EROD	362.5 ± 45.89	315.2 ± 29.00	NS	319.7 ± 8.40	NS	U/ug of total protein	NO	*F* (2,6) = 0.67	*p* = 0.5
VTG	32.14 ± 2.5	54.19 ± 3.8	**	68.34 ± 2.2	***	ng/ug of total protein	YES	*F* (2,6) = 37.82	*p* = 0.0004
CYP1A1	2.4 ± 0.62	1.01 ± 0.24	NS	0.77 ± 0.03	*	ng/ug of total protein	YES	*F* (2,6) = 5.42	*p* = 0.045
CYP11A1	0.30 ± 0.03	0.27 ± 0.02	NS	0.50 ± 0.05	*	ng/ug of total protein	YES	*F* (2,6) = 9.64	*p* = 0.013
CYP19A1	0.42 ± 0.07	0.28 ± 0.03	NS	0.77 ± 0.01	**	ng/ug of total protein	YES	*F* (2,6) = 25.38	*p* = 0.001
**Brain**									
AChE	19.48 ± 0.90	16.73 ± 0.73	*	13.5 ± 0.02	**	U/ug of total protein	YES	*F* (2,6) = 20.86	*p* = 0.002
Acetylcholine	32.24 ± 1.80	30.02 ± 1.90	NS	29.54 ± 1.20	NS	U/ug of total protein	NO	*F* (2,6) = 14.16	*p* = 0.005
Dopamine	66.80 ± 2.80	60.57 ± 2.25	NS	50.42 ± 2.80	**	pg/ug of total protein	YES	*F* (2,6) = 9.10	*p* = 0.01
Melatonin	7.91 ± 0.12	6.64 ± 0.06	**	5.64 ± 0.23	***	pg/ug of total protein	YES	*F* (2,6) = 52.11	*p* = 0.0002
GABA	0.26 ± 0.01	0.23 ± 0.00	NS	0.19 ± 0.00	**	U/ug of total protein	YES	*F* (2,6) = 19.48	*p* = 0.002
5-HT	0.85 ± 0.05	0.72 ± 0.01	NS	0.58 ± 0.03	**	ng/ug of total protein	YES	*F* (2,6) = 14.16	*p* = 0.005
Vassopressin	4.18 ± 0.23	3.28 ± 0.21	*	2.47 ± 0.13	**	ng/ug of total protein	YES	*F* (2,6) = 18.58	*p* = 0.002
Kisspeptin	11.72 ± 0.91	9.53 ± 0.18	NS	7.49 ± 0.31	**	ng/ug of total protein	YES	*F* (2,6) = 13.91	*p* = 0.005
PRL	60.45 ± 6.9	39.46 ± 6.9	NS	49.01 ± 1.9	NS	ng/ug of total protein	NO	*F* (2,6) = 3.26	*p* = 0.10
Oxytocin	24.86 ± 1.7	19.85 ± 0.17	NS	15.9 ± 1.43	**	pg/ug of total protein	YES	*F* (2,6) = 12.04	*p* = 0.007
Vasotocin	381 ± 15.29	364 ± 13.37	NS	419 ± 30.58	NS	pg/ug of total protein	NO	*F* (2,6) = 1.78	*p* = 0.24

NS, no significant; significant level was test by one-way ANOVA and post hoc analysis.

## Supplementary Materials

Supplementary Materials can be found at https://www.mdpi.com/1422-0067/21/4/1410/s1.
